# White matter development and early cognition in babies and toddlers

**DOI:** 10.1002/hbm.22488

**Published:** 2014-02-27

**Authors:** Jonathan O'Muircheartaigh, Douglas C. Dean, Cedric E. Ginestet, Lindsay Walker, Nicole Waskiewicz, Katie Lehman, Holly Dirks, Irene Piryatinsky, Sean C.L. Deoni

**Affiliations:** ^1^ Advanced Baby Imaging Lab School of Engineering, Brown University Providence Rhode Island; ^2^ Department of Neuroimaging King's College London, Institute of Psychiatry De Crespigny Park London United Kingdom; ^3^ Department of Mathematics and Statistics Boston University Massachusetts

**Keywords:** white matter, myelin volume fraction, cognitive development, multicomponent relaxometry, language, neurodevelopment

## Abstract

The normal myelination of neuronal axons is essential to neurodevelopment, allowing fast inter‐neuronal communication. The most dynamic period of myelination occurs in the first few years of life, in concert with a dramatic increase in cognitive abilities. How these processes relate, however, is still unclear. Here we aimed to use a data‐driven technique to parcellate developing white matter into regions with consistent white matter growth trajectories and investigate how these regions related to cognitive development. In a large sample of 183 children aged 3 months to 4 years, we calculated whole brain myelin volume fraction (VF_M_) maps using quantitative multicomponent relaxometry. We used spatial independent component analysis (ICA) to blindly segment these quantitative VF_M_ images into anatomically meaningful parcels with distinct developmental trajectories. We further investigated the relationship of these trajectories with standardized cognitive scores in the same children. The resulting components represented a mix of unilateral and bilateral white matter regions (e.g., cortico‐spinal tract, genu and splenium of the corpus callosum, white matter underlying the inferior frontal gyrus) as well as structured noise (misregistration, image artifact). The trajectories of these regions were associated with individual differences in cognitive abilities. Specifically, components in white matter underlying frontal and temporal cortices showed significant relationships to expressive and receptive language abilities. Many of these relationships had a significant interaction with age, with VF_M_ becoming more strongly associated with language skills with age. These data provide evidence for a changing coupling between developing myelin and cognitive development. *Hum Brain Mapp 35:4475–4487, 2014*. © **2014 The Authors. Human Brain Mapping Published by Wiley Periodicals, Inc.**

## INTRODUCTION

During postnatal development, the human brain undergoes a rapid expansion in both brain volume and cortical grey and white matter structure [Brody et al., 1987; Knickmeyer et al., 2008]. These coordinated processes provide the neural architecture underlying a dramatic development in cognitive and motor functioning. Interruption or deviation in these processes is likely to be associated with emerging childhood neurodevelopmental and psychiatric illness [Paus et al., 2008; Schumann et al., 2010; Shaw et al., 2007]. White matter maturation, and myelination in particular, has been identified as a process that may be abnormal in a variety of neurodevelopmental disorders due to its role in optimizing neuronal communication [Fields, 2008].

How this process occurs *in vivo* has remained difficult to measure. *Post‐mortem* studies dating back to the beginning of the 20th century [Flechsig, 1920] and later [Brody et al., 1987] have demonstrated a central to peripheral progression of myelination, starting in the brainstem and thalamus (*in utero*), and progressing to primary sensory and later to association cortical areas. This development is rapid in the first 2 years but progresses more slowly to as late as 30 years in humans [Fields, 2005]. While recent white matter imaging techniques, such as diffusion tensor imaging (DTI), have allowed the visualization of gross white matter architecture and is influenced by myelin content, its specificity to myelin content is limited [Jones and Cercignani, 2010]. This is most clear from studies of infants, where adult‐like patterns of fractional anisotropy (FA, a rotationally invariant metric of tissue water orientation derived from DTI) are apparent at birth, reflecting fiber architecture and coherence. Though FA values increase with age [Hermoye et al., 2006], they do not follow the same nonlinear or spatial pattern as predicted by *post mortem* studies.

An alternative *in vivo* approach, which may provide improved sensitivity to white matter microstructure and specificity to myelination, is multicomponent relaxometry [Whittall et al., 1997]. Through appropriate data acquisition and quantitative modeling of water relaxation rates, it is possible to measure the signal in tissue attributable to different water pools and specifically the water trapped between the hydrophobic layers of the myelin sheath, intra and extra cellular water and cerebrospinal fluid. The relative fraction of signal from the myelin water pool, termed the myelin water fraction (MWF), has shown strong correlations with *post mortem* myelin‐staining techniques [Laule et al., 2006, 2008] but not necessarily to DTI metrics of white matter [Kolind et al., 2008; Mädler et al., 2008], indicating that MWF may be more specific to myelin. An emerging MCR approach, multicomponent Driven Equilibrium Single Pulse Observation of *T*_1_ and *T*_2_ (mcDESPOT), offers potential advantages over conventional MCR approaches, namely decreased acquisition times, increased volumetric coverage (i.e., whole‐brain), and improved spatial resolution. Though mcDESPOT myelin water fraction measures (denoted VF_M_ to distinguish from conventional MWF) are consistently larger than corresponding MWF values, they correspond qualitatively with histological myelin content measures in an animal model of dysmyelination [Hurley et al., 2010], and reflect the disease course and clinical variability in multiple sclerosis [Kitzler et al., 2012; Kolind et al., 2012] and other demyelinating disorders [Kolind et al., 2013].

Using the mcDESPOT technique, our group has investigated the development of VF_M_ in sleeping infants and toddlers, replicating the inside‐out pattern of myelination histologically observed by Flechsig and others *in vivo* [Deoni et al., 2011, 2012b]. This work has shown a non‐linear developmental trajectory of VF_M_ from infancy to childhood, following an approximate log‐growth pattern. How this development is associated with cognitive maturation, and how individual differences may predict individual cognitive and functional ability during this period remains unclear. Previous work by our group has investigated the relationship between asymmetry of VF_M_ and cognitive abilities and indicated age‐specific relationships between subcortical and mesial frontal white matter asymmetry and language abilities [O'Muircheartaigh et al., 2013]. Contrary to expectations, no relationship was seen in asymmetry of the white matter underlying classical language areas, even though leftward asymmetry was evident in the arcuate fasciculus.

The use of multivariate techniques to interrogate large datasets of anatomical data has been increasing due to their ability to reduce high‐dimensional data into a set of meaningful spatial patterns. Although a number of methods are available, with independent component analysis [ICA, Groves et al., 2011; Li et al., 2012; O'Muircheartaigh et al., 2011; Xu et al., 2009a], principal component analysis [Narr et al., 2005] and a range of other regional covariance based techniques [Alexander‐Bloch et al., 2013a; Mechelli et al., 2005], the resulting anatomical patterns have demonstrated systems resembling those expected by regional function [Alexander‐Bloch et al., 2013b; Evans, 2013]. In addition to extracting anatomical features, these techniques can also be effective at modeling biologically uninteresting noise in the data [Xu et al., 2009a], providing improved sensitivity to anatomical changes.

A major focus of these techniques has been to investigate brain development across the lifespan, whether during childhood [Alexander‐Bloch et al., 2013b], old‐age [Bergfield et al., 2010] or across the full age range [Groves et al., 2012]. There remains a scarcity of information related to early childhood development, the period from infancy to childhood. This is in part due to the fact that the investigation of anatomical development in this age group is both technically and practically challenging [Dean et al., 2014; Raschle et al., 2012].

In this study, we focus on this overlooked developmental period by investigating the associations between mcDESPOT measures of white matter VF_M_ and cognition in a large cohort of 183 typically developing infants and toddlers. In place of using existing anatomical regions of interest, derived largely from adult datasets, we use spatial ICA to parcellate white matter from the data itself. Using the resulting regions as a base, we then test whether developmental profiles of the VF_M_ of each resulting independent component are associated with normalized cognitive scores in the same children.

## METHODS

### Participants

The Brown University institutional review board approved this study and informed consent was obtained from each participating family. One hundred and eighty three infants and toddlers (74 female) aged between 79 and 1,455 days (∼2.5 months through 4 years, corrected to a 40‐week gestation) took part in this study. Demographics of the recruited sample are shown in Table 1, split into arbitrary age groups. Inclusion criteria for this analysis were:

1. Healthy birth between 37 and 42 weeks gestation;
2. APGAR score of at least 8;
3. No reported abnormalities on fetal ultrasound;
4. No reported neurological history of the infant;
5. No major complications during pregnancy;
6. No parental self‐report of illicit drug or alcohol use during pregnancy.

**Table 1:** Demographics and cognitive summary scores

Age group (months)	*N*	Male	Female	Visual reception (SD)	Fine motor (SD)	Receptive language (SD)	Expressive language (SD)
2–12	58	39	19	48 (11.7)	50.2 (11.2)	44.2 (9.7)	45.3 (10)
12–24	64	32	32	48.5 (11)	47.6 (9.3)	44.4 (13.5)	44 (10.4)
24–48	61	38	23	57 (12.6)	47 (13.5)	53.1 (12)	52.2 (11.6)
Total sample	183	109	74	51.2 (12.4)	48.3 (11.4)	47.2 (12.5)	47.1 (11.2)

SD = standard deviation.

### Magnetic Resonance Imaging and VF_M_ Calculation

All infants and toddlers were scanned during natural sleep or, if tolerable to the child, while watching a favorite movie. The mcDESPOT multicomponent relaxometry technique was used, providing voxelwise measures of myelin volume fraction [VF_M_, Deoni et al., 2008]. Described in detail elsewhere [Deoni et al., 2008, 2012b] the mcDESPOT technique comprises a series of spoiled gradient echo (SPGR, spoiled FLASH) and balanced steady‐state free precession (bSSFP, trueFISP, FIESTA) images acquired over a range of flip angles. Acquisition parameters are selected to minimize scan time and acoustic noise (see Table 2). To complement this data, inversion recovery (IR‐) SPGR data is also acquired to correct for transmit magnetic field inhomogeneities [Deoni, 2011]. The field of view was changed according to the child's age and the image matrix was altered to provide isotropic voxel dimensions of 1.8 mm.

**Table 2:** mcDESPOT acquisition parameters by age group (Deoni et al., 2012b)

Age group	2–9 Months	9–16 Months	16–28 Months	28–48 Months
Acquisition time (min)	18:22	18:42	21:38	24:20
Field of view (cm)	14 × 14 × 13	17 × 17 × 14.4	18 × 18 × 15	20 × 20 × 15
SPGR TE/TR (ms)	5.8/12	5.9/12	5.4/12	5.2/11
SPGR flip angles	2, 3, 4, 5, 7, 9, 11, 14	2, 3, 4, 5, 7, 9, 11, 14	2, 3, 4, 5, 7, 9, 11, 14	2, 3, 4, 5, 7, 9, 12, 16
irSPGR inversion time (ms)	600 950	600 900	500 850	500 800
bSSFP TE/TR (ms)	5/10	5.1/10.2	5/10	4.4/9.8
bSSFP flip angles	9, 14, 20, 27, 34, 41, 56, 70	9, 14, 20, 27, 34, 41, 56, 70	9, 14, 20, 27, 34, 41, 56, 70	9, 14, 20, 27, 34, 41, 56, 70

irSPGR = inversion recovery spoiled gradient echo; SPGR = spoiled gradient echo; bSSFP = balanced steady state free precession; TE = echo time; TR = repetition time.

Following acquisition, mcDESPOT processing involved linear alignment of the individual flip angle images to account for subtle intra‐scan motion; removal of nonbrain signal from each image volume; calculation of main and transmit magnetic field inhomogeneities (*B*_0_ and *B*_1_, respectively); and then voxel‐wise calculation of VF_M_ through iterative fitting of a non‐linear three‐pool tissue model [Deoni et al., 2013]. Though the precision of the model fit has been questioned on the basis of unconstrained fitting parameters in a recent modeling paper [Lankford and Does, 2013], the actual fitting of mcDESPOT uses biologically constrained parameters [Deoni et al., 2013] and provides stable estimates of VFM under a range of experimental conditions [Deoni and Kolind in press].

### Cognitive Assessments

Within 1 week of successful MRI data acquisition, participants returned to be assessed using the Mullen Scales of Early Learning [Mullen, 1995]. This measure is designed to test gross and fine motor skills, visual receptive scores, and receptive and expressive language ability across a broad age range from birth to 5 years 9 months. There is a ceiling of 3 years for use of the gross motor scale so this data, where available, is not used here. The Mullen Scales have been independently normed on a representative sample of 1800 children in the US and here we use the age‐normed scores derived from this normative sample.

### Image Registration

**Figure 1:**
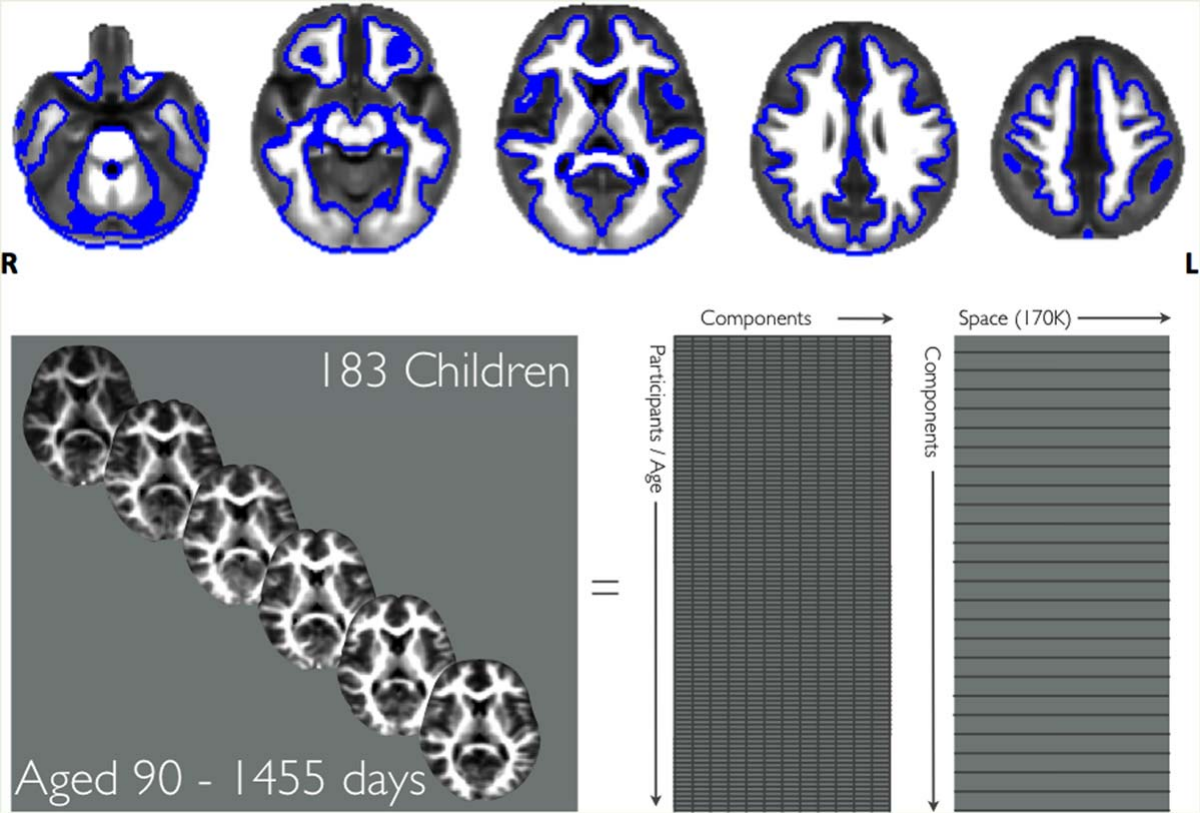
The average myelin map with the white matter mask overlaid as a blue outline. All analyses took place within this white matter mask. [Color figure can be viewed in the online issue, which is available at http://wileyonlinelibrary.com.]

The highest flip angle *T*_1_‐weighted SPGR image acquired as part of the mcDESPOT protocol for each subject (termed the reference image) was used to register each individual's VF_M_ map to a common space. The normalization procedure has been explained in detail elsewhere [Deoni et al., 2012b]. Briefly, as tissue contrast changes dramatically throughout infancy and early childhood we used a two‐stage procedure to register data to a common template image. First, the reference images were nonlinearly registered to an age appropriate template using the ANTS package [http://sourceforge.net/projects/advants/files/ANTS/, Avants et al., 2011]. A final transformation to MNI space was available from each of these age‐appropriate templates. Using these two non‐linear transformations, each participant's VF_M_ image (already in register with the reference image) was warped to template space in a single interpolation step. Once all VF_M_ images were transformed to standard space, all images were spatially smoothed using a 3D Gaussian kernel with a full‐width at half maximum of 5 mm applied within a white matter mask (3dBlurInMask, part of the afni package http://afni.nimh.nih.gov). See Figure 1 for the mask outline.

### Independent Component Analysis

The resulting spatially normalized and smoothed images were concatenated into a single 4D image and spatial ICA was performed using the melodic tool [Multivariate Exploratory Linear Decomposition into Independent Components, version 3.12, part of the fsl package http://fsl.fmrib.ox.ac.uk/Beckmann and Smith, 2004]. This probabilistic implementation of ICA automatically estimates the number of sources in the data. This leads to a set of spatially independent maps, each with a consistent temporal timecourse (i.e., growth trajectory). The resulting component spatial maps were converted to *Z*‐scores and thresholded using mixture modeling. Artifact components were visually identified and excluded from further analysis. Artifacts were identified by (a) majority of the signal occurring in edge voxels indicating misregistration (b) majority of signal occurring in areas of high cerebrospinal fluid or (c) patterns indicating motion artifact (indicated by diagonal banding throughout the component).

### Anatomical Relationships to Cognitive Scores

To investigate the cognitive relevance of the anatomical components, we performed a series of general linear models (GLM), one for each component. The GLM modeled for visual reception, fine motor skill, receptive language and expressive language ability, as well as their interactions with age. In addition, log‐transformed age and age were included as covariates of no interest. This log‐transformed age variable was included due to the predicted log‐shaped, nonlinear VF_M_ growth trajectory [Deoni et al., 2012b]. The testing of the cognition‐age interactions were included in the model to investigate the temporal stability of any cognitive relationships over the age period studied here [O'Muircheartaigh et al., 2013]. To account for multiple comparisons, we used the false discovery rate (FDR) correction [Benjamini and Hochberg, 1995] at a desired FDR rate of *q* = 0.05 (where *q* indicates the expected proportion of erroneously rejected null hypotheses among the rejected ones) and this single rate was applied across all tests.

## RESULTS

### Independent Component Analysis

**Figure 2:**
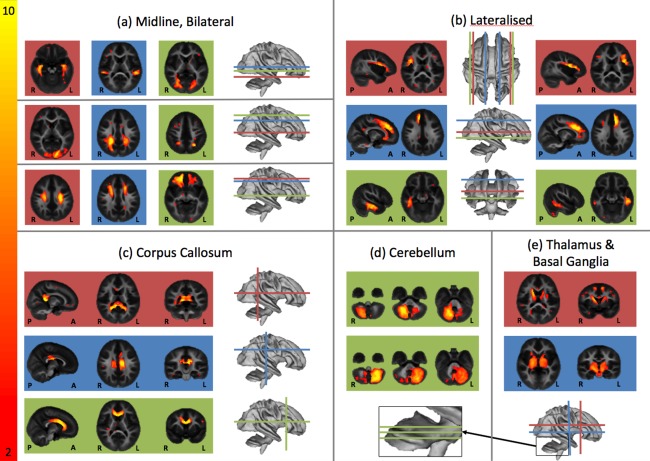
Example independent components grouped by anatomical profile. Group (**a**) demonstrates components that are bilaterally symmetric. Group (**b**) demonstrates components that are lateralized but have a symmetrical counterpart. Group (**c**) shows three aspects of the corpus callosum. Groups (**d**) and (**e**) show regions in the cerebellum, thalamus and basal ganglia respectively. Images here and throughout are presented in radiological format (left is right).

**Figure 3:**
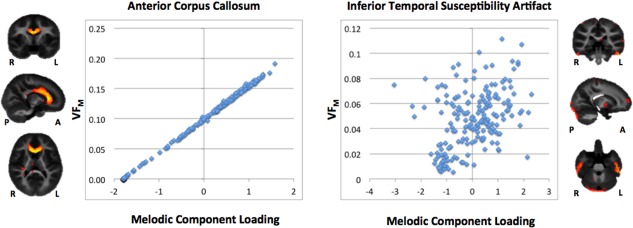
Correspondence between independent component loadings and VF_M_ in two components. The left component represents the anterior corpus callosum and shows a near 1:1 correspondence between the independent component loading and the underlying VF_M_ values. The right component's signal is predominantly in the inferior temporal lobe susceptibility area and demonstrates a less clear relationship with VF_M_.

Probabilistic ICA analysis yielded 70 spatiotemporal components. The full index of components numbered and labeled from 1 to 70 is included as Supporting Information Figure 1. Of the 70 components, 54 were identified by visual inspection as anatomically plausible unilateral and bilateral white matter bundles (e.g., thalamocortical bundles, corticocortical bundles, corpus callosum), with the remaining 16 found to be associated with structured noise (e.g., misregistration, motion artifact, nonmyelin source). Figure 2 demonstrates a subset of these components grouped by anatomical localization or spatial feature. Components that did not show a strong correspondence between component loadings and underlying VF_M_, which had most signal in edge/nonbrain voxels, or showed no correlation with age or log‐age were excluded for later analysis. Figure 3 shows examples of a likely myelin and an excluded nonmyelin component. From the plots it is clear that in the likely myelin component in the anterior corpus callosum, there is a near 1:1 correspondence between the component loading (*x*‐axis) and the calculate VFM (*y*‐axis). This relationship is nearly absent in the non‐myelin component that likely represents susceptibility artifact in the inferior temporal lobes.

### Growth Trajectories

**Figure 4:**
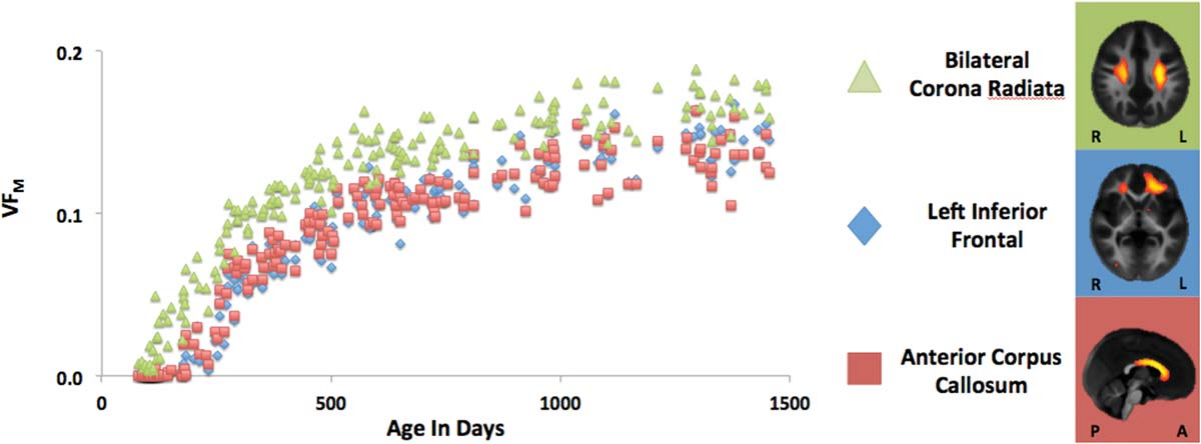
Trajectories of VF_M_ against age in the first three components. Developmental trajectories have highly nonlinear trajectory. The corticospinal tracts demonstrate a logarithmic profile and the anterior corpus callosum and left frontal white matter show a sigmoidal shape.

Population growth trajectories of the individual components (Fig. 4) showed the nonlinear growth pattern demonstrated previously in a priori regions of interest [Deoni et al., 2012b]. Although similar in their global shapes, trajectories obtained from components were different locally along the curve. For example, the component overlapping with bilateral corticospinal tract showed a rapid growth in the first year with a later slowing in rate. In contrast, two other components (corpus callosum and anterior frontal) showed a relative lag, with initial VF_M_ only becoming apparent after 200 days and then following a similar pattern to the first component, but with reduced total VF_M_. It is noteworthy that the anterior frontal component shows sustained growth compared to the other two components, given the established posterior‐to‐anterior progression of white matter maturation. The trajectories of all the independent component weightings and underlying VFM, as well as their relationship to each other, are included in Supporting Information Figures 2–7. These components are grouped grossly by focal anatomy (Supporting Information Figs. 2–5 are respectively grouped as frontal, somatosensory/parietal, occipital/temporal, subcortical/cerebellar/callosal), distributed anatomy (Supporting Information Fig. 6) or as artifact (Supporting Information Fig. 7).

### Cognitive Performance

Age‐normed *T*‐scores for the Mullen scales ranged between 47 and 51 with a standard deviation of about 11 and 12 (Table 1). As the normative sample is scaled to a mean of 50 and standard deviation of 10, we believe the sample to be representative of the general population. As expected [Mullen, 1995], there were significant correlations between the Mullen cognitive scaled scores (Table 3). The highest correlation was between receptive and expressive language scores. This is in line with the published normative data.

**Table 3:** Correlations between cognitive subscales

	Age	Gender	Visual reception	Fine motor	Receptive language	Expressive language
Age	1	−0.14	0.39^a^	−0.068	0.311^a^	0.277^a^
Gender		1	0.085	0.193	0.157	0.161
Visual reception			1	0.384^a^	0.494^a^	0.417^a^
Fine motor				1	0.377^a^	0.338^a^
Receptive language					1	0.669^a^
Expressive language						1

Correlations are Pearson's r and have 183 degrees of freedom.

a Correlation significant after controlling for multiple comparisons using Bonferroni correction.

### Cognitive Relationships With VF_M_

**Figure 5:**
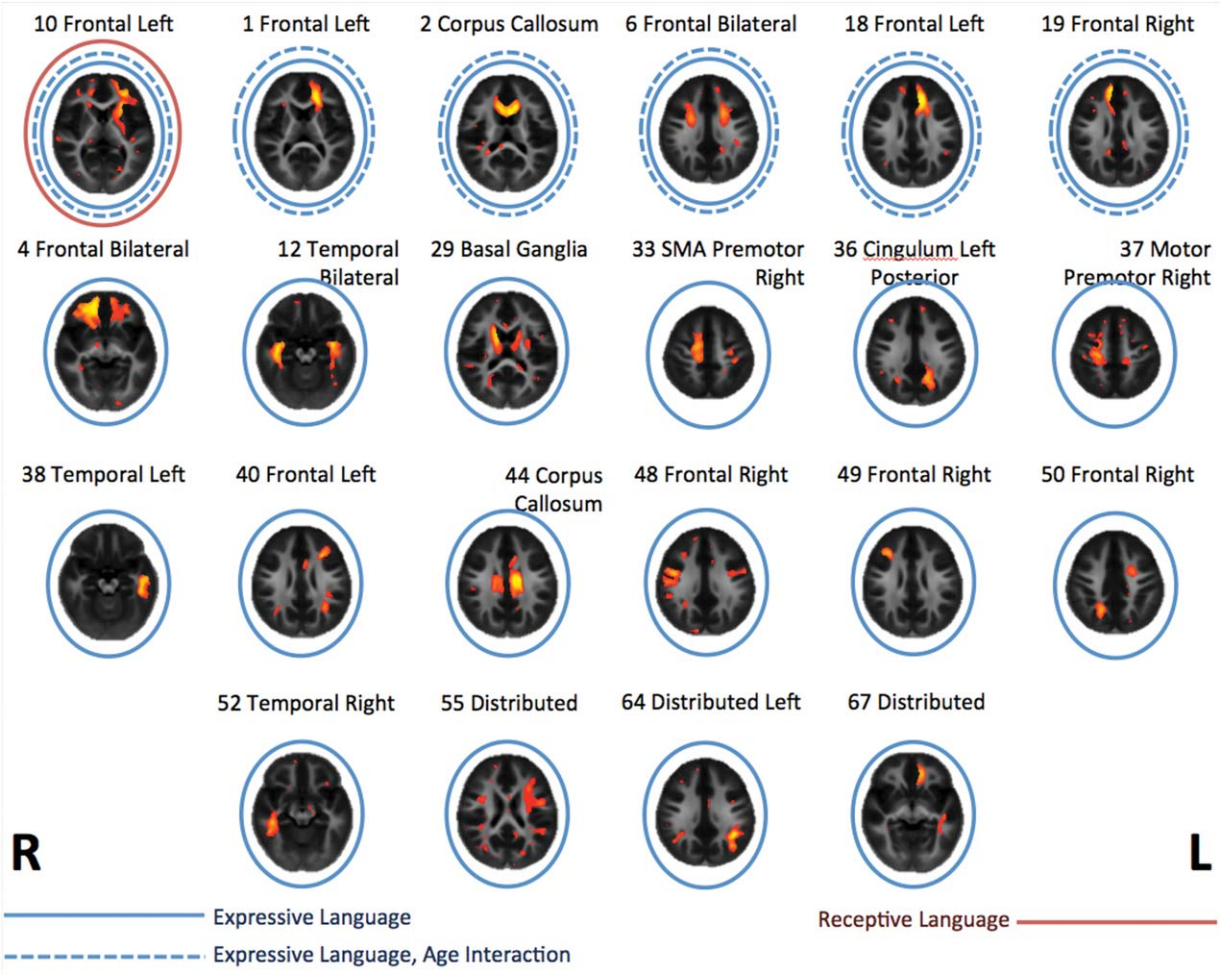
Components showing a significant relationship with receptive and expressive language scores. Red circles indicate a significant relationship with receptive language, blue with expressive language. Dashed lines indicate a significant interaction in the relationship between a component and language with age.

As would be expected in a developmental cohort, the variability of anatomically plausible components tended to be well explained by the combination of age and log‐transformed age. A subset demonstrated additional relationships with the cognitive scales. Of the 432 total contrasts of interest (54 anatomically plausible components, 4 cognitive scales, 4 age interactions with cognitive scales), there were 29 significant relationships after correction for multiple comparisons using false discovery rate (see Fig. 5).

**Figure 6:**
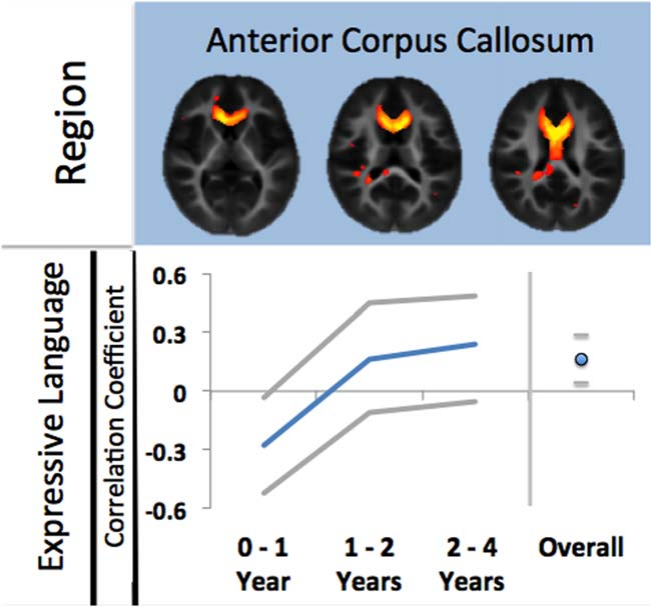
Three example regions and their linear correlations to cognitive scores. The first component in the anterior corpus callosum shows a significant relationship to both expressive and receptive language and this relationship an linear interaction with age. The second component in white matter underlying left premotor cortex shows a relationship, and interaction with age, with receptive language scores only. The third component in white matter underlying right premotor cortex shows a relationship, and interaction with age, with expressive language scores only. Note these figures are illustrative only, the statistical significance of the relationships occurs in the context of the full general linear model described in the text. Grey lines indicate the 95% confidence intervals of the correlation coefficients. [Color figure can be viewed in the online issue, which is available at http://wileyonlinelibrary.com.]

Of the four cognitive scales investigated, only the receptive and expressive language scales showed significant relationships to a subset of the components. Of the relationships with expressive language, six also exhibited significant interactions with age, indicating that the regional myelination relationships with cognitive abilities were not static over development, but rather change with age (see Fig. 6). There were no significant relationships with either fine motor or visual reception scales. Of the 22 components showing a relationship with language, 7 were left lateralized, 7 right lateralized and 8 had either bilateral representation or were midline structure. Anatomically, these components were also mostly localized in frontal (13) and temporal (3) lobes.

Importantly, it should be noted that in the context of our general linear model, the percentage variance in each component accounted for by language scales and their interactions with age was reasonably small. For example, for component 10, expressive and receptive language had an η2 value (effect size) of 0.076 and 0.049, respectively whereas the effect size of the log‐transformed age relationship was 0.619.

## DISCUSSION

Using quantitative multicomponent relaxometry MRI in a large sample of infants and toddlers, we demonstrated regional growth trajectories of VF_M_ and their associations with developing cognition. Parcellating white matter in a data‐driven way, the resulting components showed spatial correspondence with white matter bundles, sub‐cortical structures as well as bilaterally symmetrical white matter areas. These regions had distinct growth trajectories indicating their developmental relevance, with core white matter showing early and intense development and peripheral and frontal regions showing later but more sustained development, as would be predicted by histology [Fields, 2005; Flechsig, 1920; Yakovlev and Lecours, 1967]. The functional relevance of this parcellation was underlined by the regionally specific relationships with cognitive abilities in these same children. This is the first study to demonstrate this relationship with the developing myelin volume fraction. These results reinforce our recent findings demonstrating age‐specific associations between white matter asymmetry and cognition in an overlapping age group [O'Muircheartaigh et al., 2013].

The cognitive relationships were specific to language and most showed a significant interaction with age. The anatomical locations of the cognitively relevant components were predominantly in frontal regions and mostly left lateralized or bilateral (Fig. 5). White matter regions underlying classical language areas were evident as well as underlying the left middle and inferior temporal gyri, showing good correspondence with other studies investigating white matter and language in both adults [Catani, 2005] and children [Rimrodt et al., 2010]. However, associations between anatomy and cognition were more widespread. Both anterior and posterior corpus callosum (genu and splenium) as well as bilateral caudate were involved. Three components overlapped with the white matter underlying supplementary motor and premotor areas of cortex (Fig. 5, components 6, 33, 37). In addition, both left and right mesial frontal cortex was associated with expressive language only (Fig. 5, components 18 and 19), spatially consistent with an association of asymmetry in this area with language demonstrated in O'Muircheartaigh et al. [2013]. Recent work in adults has further implicated the white matter connection between midline and lateral frontal structures (the frontal aslant tract) as important in language generally, and verbal fluency specifically [Catani et al., 2013].

The lack of significant association between developing VF_M_ and nonverbal abilities may indicate that myelin content is more sensitive to verbal as opposed to nonverbal abilities in typical children. Given that visual perception and fine motor abilities may be seen as preconditions for typical language development [Iverson, 2010], it could be that these abilities do not have sufficient unique or additional information to the language scales. Importantly, relationships between typical cognitive scores and anatomy are likely to be weak. By definition, a growth curve mostly reflects age. Within the full general linear model tested here, language scores and their interactions with age accounted for ∼10% of the explained variance of the myelin data for the first component (anterior corpus callosum). Even this is likely to be an over‐estimation. Statistically, we are only controlling for differences in cognitive abilities, gender and age. Other interacting influences such as socio‐economic status [Hackman and Farah, 2009; Reilly et al., 2010], nutrition [Deoni et al., 2013], and especially genetic variation [Knickmeyer et al., [Link]] undoubtedly interact with both cognitive outcome and myelination.

How this age‐varying structural relationship corresponds to cortical and subcortical functional representation of language remains unclear. The development of language representation in babies and toddlers has been probed using functional MRI in both awake [Dehaene‐Lambertz et al., 2002] and asleep [Blasi et al., 2011] infants. A leftward asymmetry in response to language is evident as early as 2 months [Dehaene‐Lambertz et al., 2010]. These analyses indicated adult‐like functional cortical representation at an early stage in response to speech and vocalizations respectively. Importantly, in the Blasi study, there was an age‐interaction in the temporal lobe, showing increased activation in the left posterior temporal sulcus with age between 3 and 7 months. These studies are very specific to different aspects of auditory communication, but indicate a specialization of voice processing within the first year [Grossmann and Friederici, 2012].

Functional imaging in toddler groups is practically challenging. Although the use of resting‐state fMRI during sleep in young cohorts shows promise [Pierce, 2011], task based fMRI remains a challenge, in toddler groups especially. As function influences developing myelination, it is likely that early functional representation of language influences VF_M_ growth. This is a two way street. Efficient white matter pathways, indicated by VF_M_, may be critical especially during the toddler period (18–36 months). This is stage is when vocabulary goes through its largest period of growth [Ganger and Brent, 2004] and this link has explicitly been made before [e.g.. Pujol et al., 2006]. Increased communicative efficiency may result in more successful language though this can only be tested in longitudinal designs.

The use of growth trajectories to pinpoint when and where changes occur shows great promise in the elucidation of neurodevelopmental disorders [Courchesne et al., 2011; Shaw et al., 2010]. Mixed cross‐sectional longitudinal designs in very young age groups [Sadeghi et al., 2013] and older children [Lebel and Beaulieu, 2011] have demonstrated anatomical trajectories in white matter using diffusion imaging but their relationships to developing cognition are unclear. These types of mixed designs have been extremely successful in older age‐groups [Shaw et al., 2006] so this approach would likely be profitable for future research.

The age range of our data overlaps with the age‐of‐onset of a series of neurodevelopmental disorders such as autism and attention deficit hyperactivity disorder. In these disorders, morphological analysis of MRI data has identified early abnormalities in cortical structure [Courchesne et al., 2003] as well as white matter architecture [Wolff et al., 2012]. VF_M_ could provide a more specific marker of change in these populations. Myelin follows a very specific pattern of development postnatally [Brody et al., 1987], reflected here by regional trajectories, and, as we show, is related to developing cognitive ability in children. Myelin production is itself stimulated by axonal activity [Demerens et al., 1996] so abnormal neuronal functional activity may be reflected by changes in myelin patterning [Fields, 2005], something demonstrated *post‐mortem* in adults with autism [Zikopoulos and Barbas, 2010]. Longitudinal investigations of MWF in infants and toddlers at risk for neurodevelopmental disorders may allow us to detect where and when anatomical abnormalities become apparent. This approach may also provide insight on neuropsychiatric disorders typically emerging during adolescence [Paus et al., 2008].

Methodologically, the use of spatial ICA allowed us to be unbiased in our selection of regions‐of‐interest [Poldrack, 2010], allowing the data to drive the parcellation scheme. Blind source separation techniques have also been used to investigate shared features between different modalities, for example EEG and fMRI [Calhoun et al., 2009; Sui et al., 2012] or different indices of structural MRI [Groves et al., 2011, 2012; Xu et al., 2009c]. Moreover, ICA allows the separation of spatially overlapping sources [Xu et al., 2009b]. In the context of likely overlapping white matter bundles this is especially important and a significant strength in the context of studies of myelination. The modality is arbitrary for methods such as this; cognitive scores could equally be used as a feature. However, our results would suggest caution when using these methods for investigating neurodevelopment. The relationship between cognitive scores and myelin changes with age in our cohort and such a relationship would be difficult to detect using typical blind source separation techniques, instead requiring a formal model.

The age‐varying relationships, illustrated here, demonstrate the challenge in studying developmental data. Alternative functional and anatomical networks may relate to equivalent successful behavior at different stages of development [Karmiloff‐Smith, 2010; Poldrack, 2010]. This is further complicated by the difficulty in objectively measuring typical and atypical cognition and behavior in infants and toddlers. Anatomical and electrophysiological biomarkers of neurodevelopmental disorders [Bosl et al., 2011; Shaw et al., 2007] may be age‐dependent. Cross‐sectional studies that sample larger age‐ranges may be invaluable for identifying these ranges, with longitudinal studies allowing confirmation [Karmiloff‐Smith, 2010].

As has been commented elsewhere [Poldrack, 2010], there is a severe lack of research using MRI on the age range tackled here: between birth and 4 years. Though cohort studies focusing on specific ages have been instrumental in bridging this gap, the continuous range of ages used in this study covers this entire early developmental period. The population investigated here is one of the largest of its kind to date. Certainly, this constitutes the largest dataset investigating quantitative MRI in this developmental period. The age range and sampling density allows us to profile developmental trajectories during this marked period of myelin change and we have purposely sampled more densely at an earlier age range (<1 year) to accurately cover this period of intense change [Deoni et al., 2012b]. Prior studies have tended to emphasize the end of the age spectrum covered in the paper at hand [Choe et al., 2012; Knickmeyer et al., 2008] or older age groups [Evans, 2006]. Therefore, our data spans developmental periods in language and cognitive development that are not reflected in other studies.

In summary, this study demonstrates a whole‐brain white matter parcellation of the developing brain in a very large cross‐sectional sample of babies and toddlers using *in vivo* quantitative MRI collected during natural sleep. In this way, we provide a detailed view on not just developing myelin but also the white matter underpinnings of language across an under‐studied and practically difficult age‐range. These results also emphasize that relationships between structure and cognition may be age‐specific and this must be taken into account when designing developmental studies. Given the recent upsurge in interest in developmental neuroimaging as well as the role of myelin in both typical and atypical neurodevelopment, the method used here may provide an unbiased approach to interrogate trajectories of anatomical and cognitive development.

## ACKNOWLEDGMENTS

The authors thank all the families who donated their days, evenings, and precious free time to take part in this research.

## Supporting information

Supporting Information
