# Supplementary material for: White matter development and early cognition in babies and toddlers
Source: Hum Brain Mapp. 2014 Feb 27;35(9):4475–87. doi: 10.1002/hbm.22488 (PMC4336562; doi:10.1002/hbm.22488)
Supplement: Supplementary file 1 — Supporting Information [file HBM-35-4475-s001.docx]

­­

**Supplementary Figure 1:** The 70 independent components identified using spatial independent component analysis classified according to gross anatomy and laterality. Components are listed in the order of explained variance.

**Supplementary Figure 2:** Independent components localised in frontal white matter. For each component, independent component loadings (red squares) and average VF_M_ values underlying each component (blue diamonds) are plotted against age. In addition independent component loadings are plotted against VF_M_ to show their relationship.

**Supplementary Figure 3:** Independent components localised in cingulate, motor and parietal white matter. For each component, independent component loadings (red squares) and average VF_M_ values underlying each component (blue diamonds) are plotted against age. In addition independent component loadings are plotted against VF_M_ to show their relationship.

**Supplementary Figure 4:** Independent components localised in occipital and temporal white matter. For each component, independent component loadings (red squares) and average VF_M_ values underlying each component (blue diamonds) are plotted against age. In addition independent component loadings are plotted against VF_M_ to show their relationship.

**Supplementary Figure 5:** Independent components localised in cerebellar, subcortical and callosal white matter. For each component, independent component loadings (red squares) and average VF_M_ values underlying each component (blue diamonds) are plotted against age. In addition independent component loadings are plotted against VF_M_ to show their relationship.

**Supplementary Figure 5:** Independent components localised in cerebellar, subcortical and callosal white matter. For each component, independent component loadings (red squares) and average VF_M_ values underlying each component (blue diamonds) are plotted against age. In addition independent component loadings are plotted against VF_M_ to show their relationship.

**Supplementary Figure 7:** Independent components localized in non-myelinated tissue or are indicative of motion (e.g. artifact). For each component, independent component loadings (red squares) and average VF_M_ values underlying each component (blue diamonds) are plotted against age. In addition independent component loadings are plotted against VF_M_ to show their relationship.
